# Patterns of Substance Abuse in Offenders With Schizophrenia— Illness-Related or Criminal Life-Style?

**DOI:** 10.3389/fpsyt.2018.00233

**Published:** 2018-06-12

**Authors:** Thomas Stompe, Kristina Ritter, Hans Schanda

**Affiliations:** ^1^Department of Psychiatry and Psychotherapy, Medical University of Vienna, Vienna, Austria; ^2^Justizanstalt Göllersdorf, Göllersdorf, Austria; ^3^Institut für Suchtdiagnostik Wien, Vienna, Austria

**Keywords:** schizophrenia, substance abuse, alcohol, illicit drugs, criminal behavior, violence

## Abstract

**Objective:** The impact of substance abuse on violent behavior in patients suffering from schizophrenia is well-known. However, the association between the pattern of substance abuse and certain aspects of criminal behavior like the severity of offense, the previous history of violence and the age at onset of the criminal career is still unclear.

**Method:** To assess the relationship between substance abuse, schizophrenia and violent behavior we examined healthy non-offenders; healthy offenders; non-offenders suffering from schizophrenia; and offenders suffering from schizophrenia, with respect to different patterns of substance abuse (none, alcohol only, illicit drugs only, and multiple substances).

**Results:** Healthy offenders as well as offenders and non-offenders suffering from schizophrenia are characterized by increased rates of alcohol and illicit drug abuse. Especially multiple substance abuse appears to lower the threshold of aggression and illegal behavior. This effect is more pronounced in subjects suffering from schizophrenia. In both offender groups the abuse of psychoactive substances is associated with an earlier onset of the criminal career, but has no impact on the severity of the offenses.

**Conclusion:** Our results point to the need for a differentiated view on the contribution of substance abuse to the criminality of subjects suffering from schizophrenia.

## Introduction

During the last 20 years various studies have confirmed a moderate though statistically significant association between schizophrenia and violence (1–9). Most of these studies point in particular to the complex role of co-morbid substance abuse for the development of violent behavior (10–26).

However, the aforementioned association remains unclear for several reasons:

1. Compared with the general population, subjects suffering from schizophrenia exhibit significantly higher rates for substance abuse. This primarily concerns alcohol and cannabis, while opiates and hallucinogens are of minor importance (27–40). Subjects with schizophrenia and co-morbid substance abuse tend to have an earlier onset of schizophrenia than do those without co-morbidity. However, research does not support a link between specific symptoms of schizophrenia and choice of abused drugs (29).
2. Independent of a subject's mental health status, substance abuse is by itself a major criminogenic factor (41–47). A meta-analysis of 30 experimental studies confirms the common knowledge about the influence of alcohol on aggressive behavior (41).
3. Since 1996 the World Health Organization (WHO) has compiled the Global Alcohol Database to provide a standardized reference source of information for a global epidemiological survey of alcohol use and its related problems (48). Since 1999 the United Nations Office on Drugs and Crime (49) publishes the annual World Drug Report. These international health statistics clearly manifest different national prevalence rates for alcohol- and drug abuse. Additionally, interdependencies do exist between several problematic behavioral patterns such as alcohol and drug abuse, violent, and criminal behavior. The weight of these single components varies significantly in different cultures (50). Therefore, the transferability of results from American studies on the association of schizophrenia, substance abuse, and offending behavior to the European situation is limited.

Our study addresses the following questions:

- Are there differences concerning the prevalence rates and patterns of substance abuse between offenders suffering from schizophrenia and non-offenders suffering from schizophrenia?
- Are there differences concerning the prevalence rates and patterns of substance abuse between offenders with schizophrenia and healthy offenders?
- Do the rates of substance abuse in these three groups differ from the rates in healthy controls?
- Is there an association between substance abuse and patterns of criminal behavior independent of the mental status of the offenders?

## Materials and methods

### Sample

In order to assess the impact of the patterns of substances abuse on criminal behavior of male patients with schizophrenia, we used a case-control-study-design with four groups.

One hundred and three male offenders suffering from schizophrenia (OS) according to DSM-IV (51), admitted to the Justizanstalt Göllersdorf, Austria's central institution for the treatment of male mentally ill offenders not guilty by reason of insanity (NGRI).

One hundred and three healthy male offenders (OH), matched for age and severity of offense with the offenders suffering from schizophrenia.

One hundred and three male non-offenders suffering from schizophrenia according to DSM-IV (NoS) matched for age and duration of illness with the offenders NGRI.

One hundred and three male non-offenders without major mental disorders or personality disorders (NoH), matched for age with the other groups. The social stratification and the town/rural distribution of this group are representative for the overall population of Eastern Austria (52).

The protocol of the study was approved by the ethic commission of the Medical University Vienna. All patients gave written informed consent in accordance with the Declaration of Helsinki.

### Instruments

All subjects underwent the Structured Clinical Interview for DSM Disorders (SCID 1 + 2). The Inter-rater reliability tested for 25 patients was very satisfying (Cohens' alpha = 0.98). We distinguished three types of harmful substance abuse:

1. Alcohol
2. Illicit Drugs
3. Multiple substances (abuse of alcohol, benzodiazepines or illicit drugs)

The assignment to social classes was done according to Kleining and Moore (53). The pattern of criminal behavior was defined by three factors: (1) severity of the offense according to the categorical classification of Taylor (54): “minimal” (verbally aggressive; carrying a weapon which was not used; minimal accidental damage of property), “moderate” (actual bodily harm; using an offensive weapon but without causing injury; damage to property when it was the main intent), “moderately serious” (grievous bodily harm; damage to property when this was extensive and could have threatened life), and “serious” violence (life actually endangered and victim detained in hospital more than 24 h; victim died). (2) number of previous convictions, (3) age at first conviction.

### Statistics

The frequency of substance abuse was analyzed by means of Chi-Square Tests, associations between the patterns of violent behavior (severity of offense, number of previous convictions, age at onset of the criminal career) and the different types of substance abuse (none, alcohol only, illicit drugs only, multiple substance abuse) by means of One-Way ANOVA (df = 3).

## Results

The social and clinical data of the four groups are listed in Table 1. Compared to the reference group (NoH), both offender groups grew up in low social milieus. As to be expected, patients suffering from schizophrenia mostly were singles at the time of the interview (NoS) or at the time of the offense (OS). Only 4.9% of the healthy reported a life-time history of total substance abuse. In contrast both offender groups (OH, OS) showed very high rates of (comorbid) substance abuse (OH: 70%, OS: 71.8%). However, high rates were also found in the non-offending patients suffering from schizophrenia (NoS: 55.3%).

**Table 1:** Social, clinical, and offense-related data of healthy non-offenders (*n* = 103), healthy offenders (*n* = 103), non-offenders suffering from schizophrenia (*N* = 103), and offenders suffering from schizophrenia (*n* = 103).

	**NoH**	**OH**	**NoS**	**OS**
Age	33.6 ± 8.1	34.6 ± 10.4	30.8 ± 6.3	31.4 ± 9.0
**SOCIAL ORIGIN**^a^				
High	24 (23.3%)	2 (1.9%)	6 (5.8%)	5 (4.9%)
Middle	57 (55.3%)	51 (49.5%)	66 (64.1%)	38 (36.9%)
Low	22 (21.4%)	50 (48.5%)	31 (30.1%)	60 (58.3%)
Years of education	10.3 ± 8.5	7.5 ± 4.8	9.2 ± 5.7	7.1 ± 2.3
**MARITAL STATUS**				
Single	47 (45.6%)	67 (65.0%)	98 (95.1%)	91 (88.3%)
Married	47 (45.6%)	13 (12.3%)	–	2 (1.9%)
Divorced	9 (8.7%)	23 (22.3%)	5 (4.9%)	10 (9.7%)
**DIAGNOSIS**				
Schizophrenia	–	–	103 (100%)	103 (100%)
Substance abuse (total)	5 (4.9%)	69 (70%)	57 (55.3%)	74 (71.8%)
**SEVERITY OF OFFENSE**^b^				
Minimal	–	5 (4.9%)	–	3 (2.9%)
Moderate	–	24 (23.3%)	–	29 (28.2%)
Moderate serious	–	20 (19.4%)	–	18 (17.5%)
Serious	–	54 (52.4%)	–	53 (51.5%)
Number of offenses	–	4.9 ± 5.3	–	5.4 ± 5.8

NoH, healthy non-offenders; OH, healthy offenders; NoS, non-offenders suffering from schizophrenia; OS, offenders suffering from schizophrenia;

a According to Kleining and Moore (53);

b *According to Taylor (54)*.

Table 2 shows the patterns of abused substances in the four groups. Only 5 subjects of the NoH group reported periods of alcohol abuse, no single subject used illicit drugs beyond the experimental stage. In contrast, the other three groups showed statistically significantly higher rates for nearly all kinds of substance abuse. Especially the high rates of multiple substance abuse in both offending groups were striking. Interestingly, the highest prevalence of only illicit drug abuse was found in the NoS group. While the illicit drug abuse in both groups of patients suffering from schizophrenia was limited to cannabis abuse, 7.8% of the healthy offenders also reported a history of cocaine abuse and 4.8% reported opiate addiction.

**Table 2:** Patterns of lifetime substance abuse in healthy non-offenders (*n* = 103), healthy offenders (*n* = 103), non-offenders suffering from schizophrenia (*N* = 103), and offenders suffering from schizophrenia (*n* = 103).

		**NoH**	**OH**	**NoS**	**OS**
Alcohol only		5 (4.9%)	38 (36.9%)	28 (27.2%)	26 (25.2%)
	NoH				
	OH	27.3^***^			
	NoS	15.2^***^	2.2^ns^		
	OS	13.0^***^	3.3^ns^	0.1^ns^	
IlIicit drugs only		–	11 (10.7%)	26 (25.2%)	11 (10.7%)
	NoH				
	OH	11.6^***^			
	NoS	29.8^***^	7.4^**^		
	OS	11.6^***^	0.0^ns^	7.4^**^	
Multiple substances		–	20 (19.4%)	3 (2.9%)	37 (35.9%)
	NoH				
	OH	22.2^***^			
	NoS	3.0^ns^	14.1^***^		
	OS	45.1^***^	7.0^**^	35.8^***^	

NoH, healthy non-offenders; OH, healthy offenders; NoS, non-offenders suffering from schizophrenia; OS, offenders suffering from schizophrenia; Chi-Square-Tests; df = 1;

** p < 0.01;

*** *p < 0.001*.

Finally we compared the relationship between the different types of substance abuse and the patterns of criminal behavior of the two offender groups, which showed substantial similarities (Table 3). By means of One-way ANOVA we found no association between the pattern of substance abuse and the severity of the index-offense. In both offender groups subjects without any substance abuse showed the lowest number of previous convictions. In the OH group all kinds of substance abuse were associated with higher rates of previous convictions, in the OS group alcohol and multiple substance abuse—but not illicit drug abuse alone—was associated with higher rates of previous convictions. The association between psychoactive substance use and the age at first conviction was obvious in both groups. Illicit drug abuse alone or in combination with alcohol was related to earlier onset of criminal behavior.

**Table 3:** Criminal behavior and substance abuse of healthy offenders (*n* = 103) and offenders suffering from schizophrenia (*n* = 103).

	**None**	**Alcohol**	**Illicit drugs**	**Multiple**	***F***	***p***
**HEALTHY OFFENDERS**						
Severity of offense^a^	3.1 ± 0.9	3.5 ± 0.9	3.4 ± 0.9	3.2 ± 1.0	1.22	n.s.
N previous convictions	2.5 ± 2.5	5.5 ± 7.1	5.5 ± 3.9	6.7 ± 5.7	3.24	^*^
Age at 1st conviction	29.1 ± 12.7	25.9 ± 10.4	19.1 ± 9.1	19.1 ± 4.9	5.24	^**^
**OFFENDERS SUFFERING FROM SCHIZOPHRENIA**						
Severity of offense^a^	3.4 ± 0.9	3.2 ± 0.8	3.0 ± 0.8	2.9 ± 1.0	2.12	n.s.
N previous convictions	2.9 ± 3.6	4.9 ± 5.7	2.6 ± 1.4	8.5 ± 6.6	7.61	^***^
Age at 1st conviction	28.9 ± 11.3	26.2 ± 9.9	21.4 ± 5.1	21.4 ± 7.2	4.19	^**^

a According to (54); One-Way ANOVA; df = 3;

* p < 0.05;

** p < 0.01;

*** *p < 0.001*.

## Discussion

Comparison of the four groups shows that a statistically significantly higher prevalence of substance abuse is associated with both psychiatric illness and offending behavior (Table 2). Only 4.9% of the healthy controls reported a history of alcohol abuse, which is lower than the prevalence rates reported in studies by WHO (48) and UNODC (49). This may be due to the fact that one of the inclusion criteria for the control group was the absence of a DSM-IV Axis I disorder and, especially, of any personality disorder.

Non-offending patients with schizophrenia differ from the other groups by a relatively high prevalence of illicit drug abuse (25.2%) which is nearly exclusively confined to cannabis. This is in line with previous findings of studies on the use of cannabis in schizophrenic patients (40, 55, 56). During the last 20 years three main hypotheses were put forward to explain the increased cannabis use of patients with schizophrenia: (a) cannabis abuse triggers the outbreak of schizophrenia (57), (b) cannabis abuse follows the outbreak of schizophrenia (patients claim improved well-being and coping with stress, facilitation of social interaction, improvement of depression, better control of psychotic symptoms, and reduction of the side-effects of the anti-psychotic medication), and (c) cannabis abuse and schizophrenia reinforce each other.

Compared with the offenders suffering from schizophrenia, significantly fewer cases of multiple substance abuse were identified among the non-offenders suffering from schizophrenia (35.9 vs. 2.9%; Table 2). In addition, the patterns of substance use varied to a large extent: while all of the mentally ill offenders consumed alcohol in addition to the abuse of cannabis or benzodiazepines, the illicit substance abuse of five of the healthy offenders also included opiates or cocaine. While illicit substance abuse alone was not a strong predictor of illegal behavior, the combination of illicit drugs and alcohol appears to stimulate criminal behavior especially in patients suffering from schizophrenia. This result is in line with studies pointing to an association between alcohol abuse and aggressive behavior in mentally ill as well as in healthy subjects (42, 57, 58). According to Giancola and Parrott (44), alcohol significantly strengthens the relation between stimulant drug use and aggression among men. The increased aggressiveness under alcohol is probably caused by three interacting mechanisms (59): (a) disinhibition, (b) aggravation of aggressive impulses, and (c) behavioral disorganization.

Although our data did not suggest a link between cannabis use and criminal behavior in general, we must not underestimate other problematic consequences of cannabis abuse such as non-compliance, persistence of psychotic symptoms, and reinforcement of negative symptoms (58).

The influence of substance abuse on the pattern of criminal behavior was very similar in healthy and schizophrenic offenders (Table 3). Our results lead to the conclusion that alcohol and illicit drugs have no influence on the severity of offenses. However, we found a clear association between any kind of substance abuse and the frequency of criminal acting as well as a significant relationship between illicit drugs and an early start of the criminal career, independent of the mental health status of the subjects (60). Our data suggest that the pattern of substance abuse by schizophrenic offenders shows more similarities to that of healthy offenders than to that of non-offenders with schizophrenia. These findings are in line with our own study on family structures and socialization (see also Table 1) (61, 62). Independent of the mental state, dysfunctional family structures and substance abuse are associated with illegal conduct. However, our data must not be understood as an assertion that schizophrenia itself does not account for criminal behavior at all. Although certain psychotic features of schizophrenia, as for example threat-control-override symptoms are not very important for criminal behavior of psychotic patients in general, they are strongly associated with severe aggressive acts, such as severe bodily injury and homicide (63).

## Main findings

In accordance with the literature, high rates of substance abuse in general are associated with both, schizophrenia and offending behavior.

High rates of only illicit substance abuse—especially cannabis—are also found among patients suffering from schizophrenia without any history of offending behavior.

However, the abuse of illicit substances combined with alcohol and the abuse of alcohol only are associated with high rates of criminal behavior among patients with schizophrenia. In particular, the abuse of illicit drugs was found in a subgroup with an early onset of criminal behavior.

## Strengths and limitations

The sample size of the study (103 mentally disorders NGRI and 3 matched control groups) allows to draw robust and differentiated conclusions concerning the impact of substance abuse on criminal behavior in offenders suffering from schizophrenia. However, Eisner (50) stated that the associations between the single features of problematic conduct such as violent and illegal behavior, alcohol and illicit drug use, show substantial cultural variations.

Therefore we have to take into account the globally different rates of criminality and substance abuse. As they are substantially higher in the USA compared to Europe, the results of US-studies apply only partly to European countries. Austria with its low rates of criminality and substance abuse (except alcohol) is in line with most of the other Western European countries. As a consequence, methodologically similar studies in other regions are necessary to investigate the variations of the impact of the culture-specific types of substance consumption on the criminal behavior of patients suffering from schizophrenia.

## Author contributions

TS and HS planned the study and collected the data of the offender groups. TS did the statics and wrote the paper. KR collected the data of both non-offending groups.

### Conflict of interest statement

The authors declare that the research was conducted in the absence of any commercial or financial relationships that could be construed as a potential conflict of interest.
